# Androgen Receptor Accelerates Premature Senescence of Human Dermal Papilla Cells in Association with DNA Damage

**DOI:** 10.1371/journal.pone.0079434

**Published:** 2013-11-14

**Authors:** Yi-Chien Yang, Hung-Chun Fu, Ching-Yuan Wu, Kuo-Ting Wei, Ko-En Huang, Hong-Yo Kang

**Affiliations:** 1 Department of Dermatology, Kaohsiung Chang Gung Memorial Hospital, Kaohsiung, Taiwan; 2 Department of Obstetrics and Gynecology, Kaohsiung Chang Gung Memorial Hospital, Kaohsiung, Taiwan; 3 Department of Chinese Medicine, Chang Gung Memorial Hospital, Chiayi, Taiwan; 4 Graduate Institute of Clinical Medical Sciences, Chang Gung University, College of Medicine, Kaohsiung, Taiwan; 5 Center for Menopause and Reproductive Research, Kaohsiung Chang Gung Memorial Hospital, Kaohsiung, Taiwan; Columbia University Medical Center, United States of America

## Abstract

The dermal papilla, located in the hair follicle, expresses androgen receptor and plays an important role in hair growth. Androgen/Androgen receptor actions have been implicated in the pathogenesis of androgenetic alopecia, but the exact mechanism is not well known. Recent studies suggest that balding dermal papilla cells exhibit premature senescence, upregulation of p16*^INK4a^*, and nuclear expression of DNA damage markers. To investigate whether androgen/AR signaling influences the premature senescence of dermal papilla cells, we first compared frontal scalp dermal papilla cells of androgenetic alopecia patients with matched normal controls and observed that premature senescence is more prominent in the dermal papilla cells of androgenetic alopecia patients. Exposure of androgen induced premature senescence in dermal papilla cells from non-balding frontal and transitional zone of balding scalp follicles but not in beard follicles. Overexpression of the AR promoted androgen-induced premature senescence in association with p16*^INK4a^* upregulation, whereas knockdown of the androgen receptor diminished the effects of androgen. An analysis of γ-H2AX expression in response to androgen/androgen receptor signaling suggested that DNA damage contributes to androgen/androgen receptor-accelerated premature senescence. These results define androgen/androgen receptor signaling as an accelerator of premature senescence in dermal papilla cells and suggest that the androgen/androgen receptor-mediated DNA damage-p16*^INK4a^* axis is a potential therapeutic target in the treatment of androgenetic alopecia.

## Introduction

Androgenetic alopecia (AGA) is an androgen-mediated disorder that causes hair thinning in a defined pattern [1], [2]. The prevalence of AGA increases with aging, from 31% at age 40–55 years to 53% at age 65–69 years [3]. However, the pathogenetic mechanisms underlying AGA are not fully understood. The skin and the pilosebaceous unit are androgen target tissues. In dermal papilla cells (DPCs), the major circulating androgen, testosterone (T), can be locally metabolized to dihydrotestosterone (DHT) by steroid 5α-reductase. Based on its affinity for binding to the androgen receptor (AR), DHT is ten-fold more potent than testosterone [4]. According to the observation that male subjects with genetic deficiency of type 2 5α-reductase do not develop scalp hair loss [5], DHT has been suggested to be a major determinant in the pathophysiology of AGA in men. In AGA, DPCs from androgen-sensitive frontal scalp contain more AR and steroid 5α-reductase than those from androgen-insensitive occipital scalp [6]. AGA is characterized by miniaturization of hair follicles in the androgen-sensitive frontal scalp. The volume of dermal papilla (DP) depends on its number of DPCs and the amount of extracellular matrix, and correlates with the size of the hair fiber produced [7]. Whereas previous studies have shown that androgens do not alter the proliferation of DPCs [8], [9]. In clinical observations, blocking the conversion of T to DHT with finasteride, a 5α reductase inhibitor, does not reverse miniaturized follicles to thick hair fibers in advanced AGA [10], suggesting that androgens/AR might irreversibly cause the damage of hair follicles.

Premature senescence is thought to accelerate the appearance of senescent phenotypes in cells upon exposure to sublethal stressors [11]. Several studies have shown that senescent cells are morphologically altered (enlarged and flattened) [12], and express more extracellular matrix-degrading proteases, collagenase, and matrix metalloproteinases [13], [14]. In addition to its ability to deplete the renewal capacity of tissues by causing proliferative arrest, senescence may contribute to aging by influencing neighboring cells be means of secretory molecules thus disrupting the integrity and homeostasis of tissues [15]–[17]. A recent study indicated that balding DPCs undergo premature senescence in vitro in association with expression of senescence-associated β-galactosidase (SA β-gal) and p16*^INK4a^* protein, and markers of oxidative and DNA damage [18]. It has been known that the DNA-damage response is a central mediator in triggering cellular senescence [19] and androgens act as DNA-damaging agents that generate DNA double-strained breaks (DSBs) and thus facilitate chromosomal translocation in androgen-sensitive prostate cancer cell lines [20]. However, how androgen/AR signaling leads to DNA damage in DPCs has not been elucidated.

To examine whether androgens may contribute to premature senescence by promoting DNA damage in DPCs of AGA patients, we cultured DPCs obtained from the frontal scalp of AGA and non-AGA individuals and determined their senescence phenotypes and investigated the effects of androgen/AR signaling on the development of premature senescence. We also studied the p16*^INK4a^* protein and the relationship between androgen/AR signaling and DNA damage markers to elucidate the possible mechanisms of androgen/AR-accelerated premature senescence in DPCs.

## Materials and Methods

### Ethics Statements

The Chang Gung Medical Foundation institutional review board approved all described studies (protocol number 99-1933B). The study was conducted according to the Declaration of Helsinki Principles. Informed written consent was obtained from all patients.

### Isolation and Culture of Human DPCs

Specimens were taken from beard, transitional zone of balding, balding or non-balding frontal scalps of ten males undergoing surgical excision of benign cutaneous tumors. The donors of beard specimen were aged 28 and 34 years, and the donor of transitional zone of balding scalp was aged 38 and 45 years. Four were AGA patients aged 20, 24, 27 and 40 years, and the others were age-matched (20, 24, 27 and 40 years) non-AGA individuals. DPs were isolated from the bulbs of dissected hair follicles, transferred onto plastic dishes coated with 0.1% gelatin, and cultivated in Dulbecco’s modified Eagle’s medium (DMEM) containing 10% fetal bovine serum (FBS), 100 IU/mL penicillin/100 µg/mL streptomycin and 0.4 mM L-glutamine (Sigma) in a humidified 95% atmosphere with 5% CO_2_ at 37°C. DPCs from the second to sixth passages of subcultures were used.

### SA β-gal Staining

The SA-β-Gal activity was determined by using a SA-β-Gal staining kit (Sigma). SA-β-Gal staining was detected in cultured DPCs seeded at a density of 10^5^ cells in six-well plates and frozen human hair follicles in slide-mounted sections.

### Measurement of Cell Size

Photomicrographs of DPCs were captured using a Nikon TE300 microscope equipped with a Nikon DSFi1 camera (Nikon Japan), and cell size was analyzed with Image-Pro Plus 6.1 software.

### Cell Doubling Time Estimation

DPCs were trypsinized in exponential growth phase. Equal numbers of cells (1×10^4^) were plated in six-well culture plates (day 0) and cultured in 5% CO2 at 37°C. Viable cells were determined by the trypan blue exclusion test and counted in triplicate on days 1, 3, 5. Cell doubling time, DT, was estimated by the following formula: DT = (t1–t0 )log 2/(log N1−log N0 ) where N1 and N0 are the number of cultured cells at the current (t1) and previous (t0) measurements.

### Co-culture of DPCs and Keratinocytes (KCs)

Primary human hair follicular KCs were purchased and maintained in keratinocyte growth medium (KGM) (ScienCell research laboratories, California, USA). For the experiment, KCs in the third to fourth passage of subculture were used. DPCs in the second passage were treated with or without 0.1 µM of DHT in 10 cm culture dishes for 5 days. Then DPCs were seeded at a density of 5×10^4^ cells/well in the lower compartment of transwell multiplates (six-wells, Corning, NY, USA). At the same time, KCs (5×10^4^ cells/well) were also seeded with KGM into the transwell inserts with type I and III collagen–coated (24 mm diameter, 0.4 µm pore size, Corning). After incubation overnight, the medium was changed to MCDB153 (Sigma) without growth factors and bovine pituitary extract and DPCs and follicular KCs were cocultured and 0.1 µM of DHT or ethanol was added to these cultures. After they were incubated for 4 days, we counted the number of KCs in each well.

### Transfection of DPCs

The AR expression plasmid, pcDNA3–hAR [21], [22], pcDNA3–hERα [22] has been described previously. For overexpression of the AR or estrogen receptor α (ERα), DPCs were transiently transfected with pcDNA3–hAR or pcDNA3–hERα using the calcium phosphate method. AR expression was knocked down by infection with lentiviral vector pLKO.1-puro carrying a small hairpin RNA (shRNA) specific for AR-shRNA (5′-CACCAATGTCAACTCCAGGAT-3′). Reagents for RNA interference (RNAi) experiments were acquired from the National RNAi Core Facility at the Institute of Molecular Biology/Genomic Research Center, Academia Sinica, Taiwan. Infected cells were selected by incubating with puromycin (2 µg/mL; Sigma) for 4 days. Cells infected with a scrambled shRNA (5′-TCAGTTAACCACTTTTT-3′) were used as a control.

### Immunofluorescence Staining of Phospho-(Ser-139)-H2AX (γ-H2AX)

DPCs were plated in eight-chamber slides (Nunc Lab-Tek, Roskilde, Denmark) at a density of 3×10^3^ cells per well. Immunofluorescence staining of γ-H2AX was performed as previously described [23]. Briefly, proteins were immunolabeled by incubating with anti-γ-H2AX antibody (1∶200; Cell Signaling, Danvers, MA, USA) and goat anti-rabbit Alexa Fluor 568-conjugated antibody (1∶500; Invitrogen, OR, USA). Slides were examined under a Nikon E800 microscope, and images were collected using a SPOT RT3 microscope camera. Captured images were analyzed for foci using AlphaEase FC software, and foci were quantified as described previously [24], [25].

### Detection of Senescence Associated Heterochromatin Foci (SAHF)

DPCs were seeded onto eight-chamber slide at a density of 3×10^3^ cells per well. After fixing cells with cold methanol, SAHF were detected by staining with 0.13 µg/mL 4′,6-diamidino-2-phenylindol (DAPI) for 2 minutes at room temperature as described [26], and foci-positive DPCs were counted.

### Western Blot Analysis

Cells were lysed in lysis buffer supplemented with 1% protease inhibitor cocktail (Roche Applied Science, Indianapolis, IN, USA). Proteins in whole-cell lysates were resolved by sodium dodecyl sulfate-polyacrylamide gel electrophoresis and transferred to polyvinylidene fluoride (PVDF) membranes. Membranes were probed with polyclonal rabbit anti-AR primary antibody (N-20 1:500; Santa Cruz Biotechnology, Inc., Carpinteria, CA, USA), anti-ERα primary antibody (1∶400; Millipore Corporation, Billerica, MA, USA) anti-p16 antibody (1∶200; Santa Cruz Biotechnology) or anti-γ-H2AX antibody (1∶1000; Cell Signaling), and then incubated with horseradish peroxidase-conjugated secondary antisera (Amersham, Buckinghamshire, UK). Enhanced chemiluminescence was performed with ECL-Plus (Amersham Pharmacia ), and bands were quantified by densitometry using ImageJ software (US National Institutes of Health, Bethesda, MD, USA).

### Statistical Analysis

All values are presented as means ± standard deviations (SDs) of replicate samples, and experiments were repeated a minimum of three times. Differences between two groups were assessed using unpaired two-tailed Student’s t test. In all statistical comparisons, *P*<0.05 was defined as significant. SPSS statistics software (Version 15.0) was used for all calculations.

## Results

### Balding DPCs from AGA Patients are more Senescent than Non-balding DPCs

**Figure 1:**
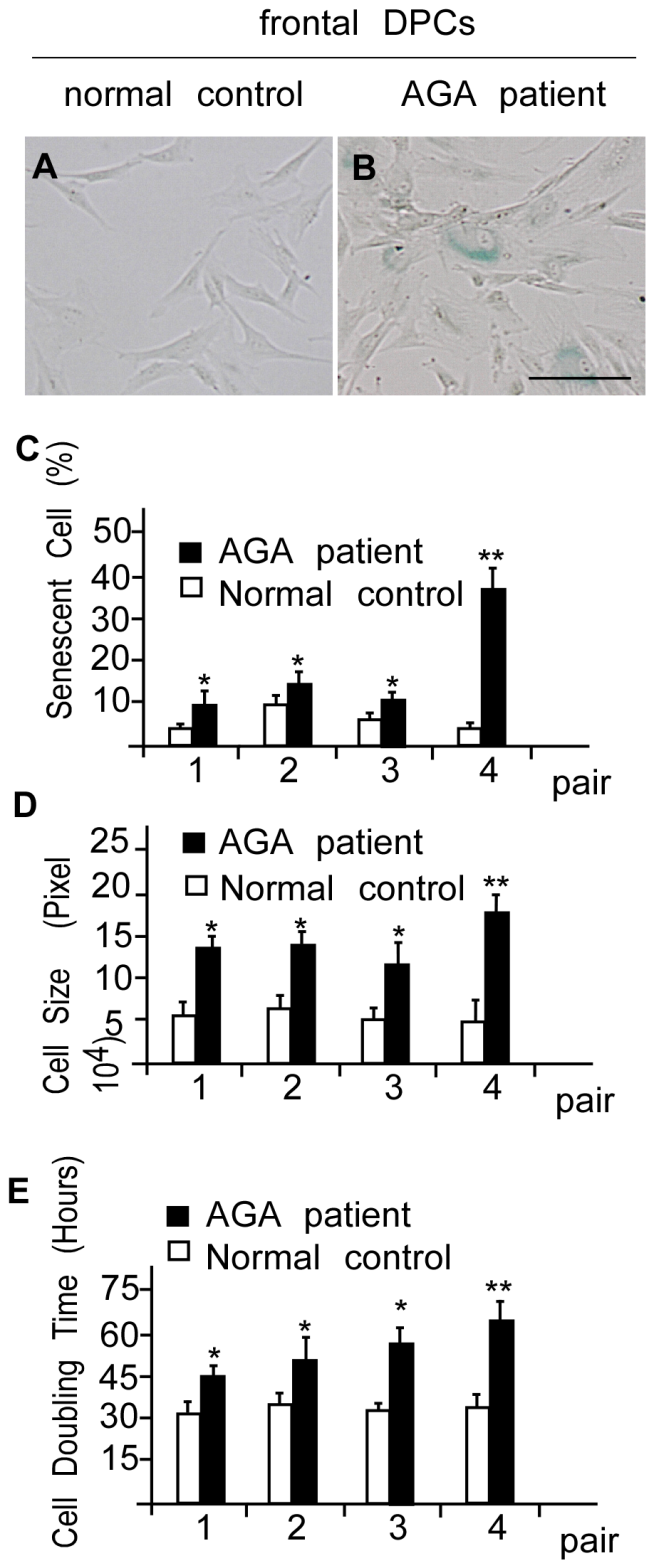
Balding DPCs are more senescent than sex-, age-, and site-matched non-balding DPCs. (A) Non-balding DPCs isolated from the frontal scalp of normal individuals exhibited a relatively normal appearance at passage 2 compared with balding DPCs isolated from the frontal scalp of AGA patients of the same passage, which exhibited an enlarged, irregular, and flattened morphology. (B) SA-β-Gal activity was increased in balding DPCs. Scale bar = 100 µm. (C) Quantification of SA-β-Gal activity showed that the percentage of SA-β-Gal expression was increased in balding DPCs in all matched-pairs. (D) Balding DPCs exhibited an increase in cell size. (E) Prolongation of the cell doubling time were observed in balding DPCs. The pair 1–4 of the x axis in Figure 1C,D,E means the each age (20, 24, 27 and 40 years), sex (male), site (frontal) matched pairs of normal control (non-AGA males) and AGA patient. Values are means ± SDs from three determinations per experiment from three independent experiments using second-passage DPCs (*P<0.05, **P<0.01 compared with matched controls).

A previous study has shown that DPCs from balding (frontal, androgen sensitive) scalps of AGA patients display a more premature-senescence phenotype than DPCs from non-balding (occipital, androgen insensitive) scalps of AGA patients [18]. To further compare senescence phenotypes between balding and non-balding DPCs isolated from androgen-sensitive frontal scalps, we harvested DPCs from frontal scalps of eight males (four AGA patients with frontal baldness and the others were age-matched normal individuals) and stained second-passage primary DPCs for SA-β-Gal activity. Balding DPCs isolated from AGA patients showed broader and polygon-shaped morphology (Fig. 1B) in contrast with the elongated, fibroblast-like appearance of DPCs isolated from the normal individuals (Fig. 1A). We also observed stronger positive SA-β-Gal staining and larger cell size in balding DPCs than non-balding DPCs of the same passage (Fig. 1C and D). The average of cell doubling time of balding DPCs was 56.3 hours compared to 32.5 hours of the non-balding DPCs. While the cell doubling time was variable among the same passage of balding DPCs from different AGA patients, the balding DPCs have relative longer cell doubling time compared to non-balding DPCs (Fig. 1E).

### Androgen Treatment Promotes Senescence in Earlier-passage DPCs from Frontal Scalp

**Figure 2:**
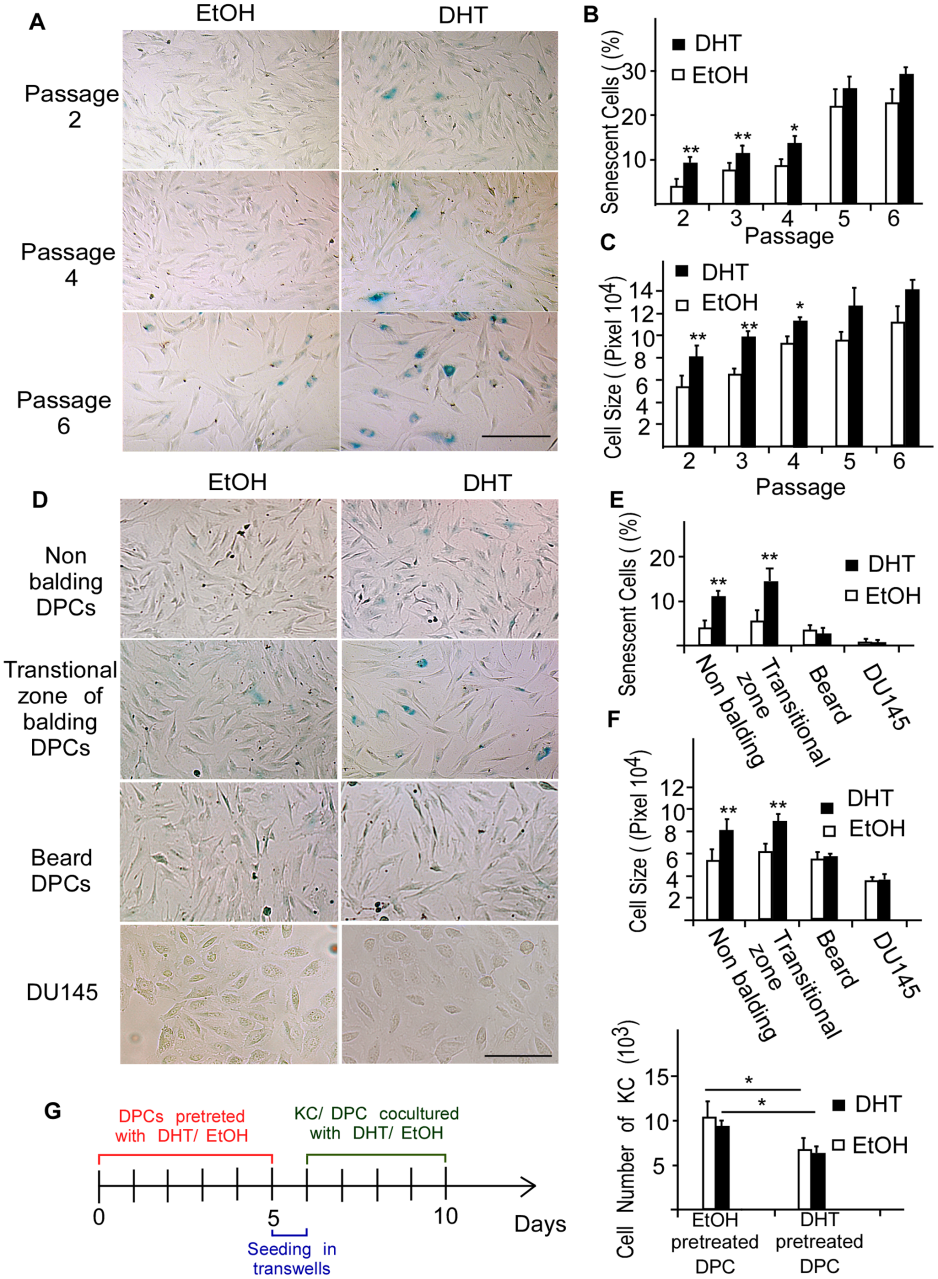
Androgen promotes senescence in earlier-passage DPCs of frontal scalp. DPCs of various origin and DU145 cells were treated with 0.1 µM of DHT for 3 days and then stained for SA-β-Gal 5 days after DHT stimulation. (A–C) Early and late passages of non-balding DPCs are shown for comparison. SA-β-Gal activity increased after DHT stimulation. A quantitative analysis revealed a significant increase in the percentage of senescent DPCs in DHT-treated, earlier-passage groups. DHT-induced premature senescence was also evaluated by measuring cell size. The bar graph shows quantification results. (D–F) DHT accelerated premature senescence in non-balding and transitional zone of balding DPCs only. Scale bar = 200 µm. Values are means ± SDs from three independent experiments (*P<0.05, **P<0.01 compared with cells in vehicle-containing medium). Each data resulted from primary culture cells from 3 individuals and each was measured in triplicates in each cell line. In Figure 2D (transitional zone of balding scalp and beard), we used primary culture cells from 2 individuals with three determinations per experiment from 3 independent experiments. (G) The functional defect of DHT-mediated senescent DPCs in the interaction with hair follicular KCs. Time scale of the experiment and the cell numbers of KC cocultured with DPCs were shown. Values are means ± SDs from three independent experiments. Each data resulted from primary culture cells from 3 individuals and each was measured in triplicates in each cell line. Asterisk indicates *P*<0.05.

To determine whether the senescence phenotypes in DPCs of different passages were differentially affected by androgen treatment, non-balding DPCs isolated from frontal scalp of normal individuals were cultured in the absence or presence of 0.1 µM of DHT used in previous AGA studies [27], [28]. We found that non-balding DPCs of various passages exhibited increased SA-β-Gal activity compared with vehicle (ethanol)–treated cells (Fig. 2A). Quantification of these results revealed a significant increase in the percentage of SA-β-Gal–positive cells after DHT stimulation (Fig. 2B). Moreover, cells exposed to DHT were increased in size and had broader morphology. DHT-induced premature senescence was more significant at earlier passages: in second- and third-passage cells, the premature senescence phenotype, including positive-SA-β-Gal staining and cell-size enlargement, both reached statistical significance (Fig. 2B, C). Although more DPCs exhibited senescence phenotypes and induction of premature senescence by DHT persisted in passages four to six, statistical significance was gradually lost. To further investigate the linkage between the DHT-accelerated premature senescence of DPCs and the pathogenesis of AGA, we compared the DHT effects on premature senescence of DPCs isolated from transitional zones of balding scalp, beard, and androgen-unresponsive human prostate cancer cell line, DU145. We found that DHT induced premature senescence significantly in the DPCs from both transitional zone of balding scalp and non-balding frontal scalp. In contrast, neither beard DPCs (passage 2) nor DU145 cells became senescent after exposed to DHT 0.1 µM (Fig. 2D, E and F). To access the functional defect of DHT-mediated senescent DPCs in the interaction with hair follicular KCs, we employed an *in vitro* co-culture system [29]. DPCs was first cultured in the presence or absence of 0.1 µM of DHT for 5 days to induce premature senescence and then co-cultured with follicular KCs for 4 days in the presence or absence of DHT. As shown in Fig. 2G, the growth of KCs cocultured with DHT-pretreated DPCs was decreased. Together, we suggest that DHT-accelerated premature senescence is a specific action in earlier-passage DPCs from frontal scalp and DHT-mediated senescent DPCs may have the functional defect to communicate with KCs and play an important role in AGA pathophysiology.

### AR is Required for Androgen-induced Premature Senescence in DPCs

**Figure 3:**
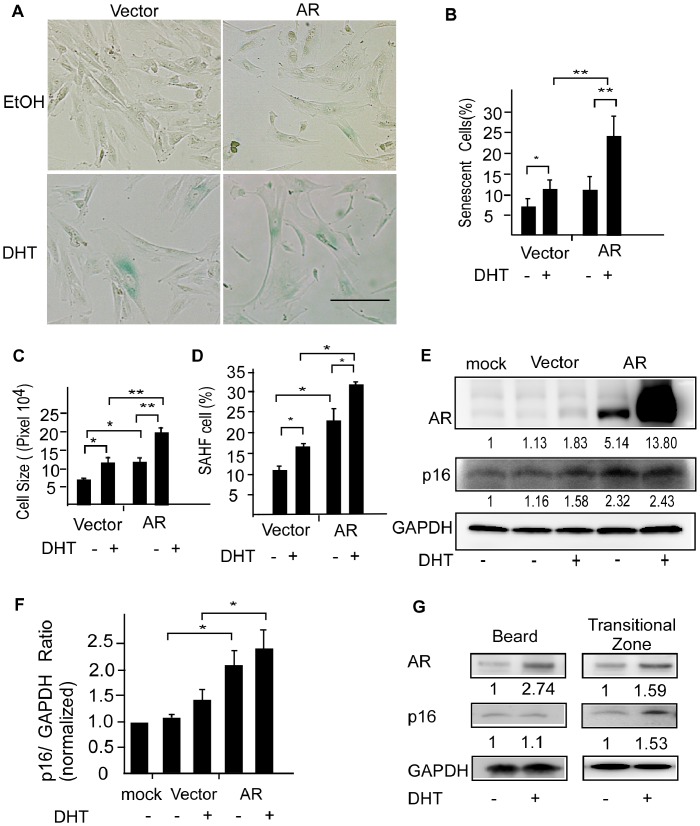
Overexpression of the AR promotes androgen-accelerated premature senescence in DPCs. (A) Non-balding DPCs of frontal scalp were transfected with pcDNA3-hAR, or pcDNA3 empty vector and cultured in the presence of DHT or ethanol (vehicle control) for 3 days. Premature senescence of DPCs was evaluated on day 5. Scale bar = 100 µm. DHT increased SA-β-Gal activity (B), cell size (C), and the number of SAHF-containing DPCs. (D) Overexpression of AR enhanced the statistical significance of DHT effects. Values are means ± SDs from three independent experiments (*P<0.01, **P<0.001). (E)A representative immunoblot of cell lysates of DPCs after treatment with DHT or vehicle for 84 hours and (F) Quantitative densitometry of the p16/GAPDH protein expression was performed by using ImageJ software. Values are means ± SDs from 6 independent experiments Asterisk indicates *P*<0.05. (G) DHT increased p16*^INK4a^* protein expression in DPCs isolated from transitional zone of balding scalp but unaltered in DPCs isolated from beard. The numbers indicate p16*^INK4a^*/GAPDH and AR/GAPDH ratios. GAPDH (glyceraldehyde 3-phosphate dehydrogenase) was used as an internal standard.

To further confirm the role of AR expression in androgen-accelerated premature senescence of DPCs and mimic balding DPCs, which contain higher levels of AR, we manipulated AR expression levels in DPCs. Non-balding DPCs isolated from the frontal scalp were transfected with an AR expression plasmid or vector control in the absence or presence of 0.1 µM of DHT. Premature senescence of DPCs was analyzed by staining for SA-β-Gal and measuring cell size. Overexpression of the AR increased both percentage of SA-β-Gal–positive cells and cell size and DHT enhanced the AR effects (Fig. 3A–C). To firmly establish the relationship of the AR with the androgen-induced senescence phenotype of DPCs, we examined SAHF, a nuclear marker of senescence characterized by punctuate intranuclear foci in DAPI–stained cells. A quantitative analysis showed that DHT induced SAHF formation in DPCs, and overexpression of the AR reinforced the effects of DHT compared with vector control-transfected cells (Fig. 3D). To gain insight into the mechanism of premature senescence induction in DPCs, we focused on the relationship between the AR and p16*^INK4a^* protein, which is known to be involved in premature senescence and is upregulated in balding DPCs [18]. Although we saw an increase in DHT-induced p16 protein expression in DPCs isolated from the frontal scalp, quantification of these data showed that the effect of DHT on p16 expression was not significantly different and only p16 levels in DPCs with AR overexpression compared with empty vector cell were statistically significant (Fig. 3E, F). The p16*^INK4a^* protein is also up-regulated in DHT-treated DPCs isolated from transitional zone of balding scalp but unaltered in DPCs isolated from beard (Fig. 3G). This indicates that androgens/AR signaling is functionally linked to premature senescence that is region-specific phenomenon (androgen sensitive scalp) in DPCs and associated with the pathogenesis of AGA. One of the important considerations of premature senescence in DPCs is whether this is a specific androgen/AR action. Thus, ERα was employed as a negative control in these experiments. Increasing percentage of SA-β-Gal–positive cells, cell size, SAHF formation and induction of p16*^INK4a^* protein levels were not observed in DPCs overexpressing ERα with 0.01 µM of 17β-estradiol stimulation (Supplemental Figure 1).

**Figure 4:**
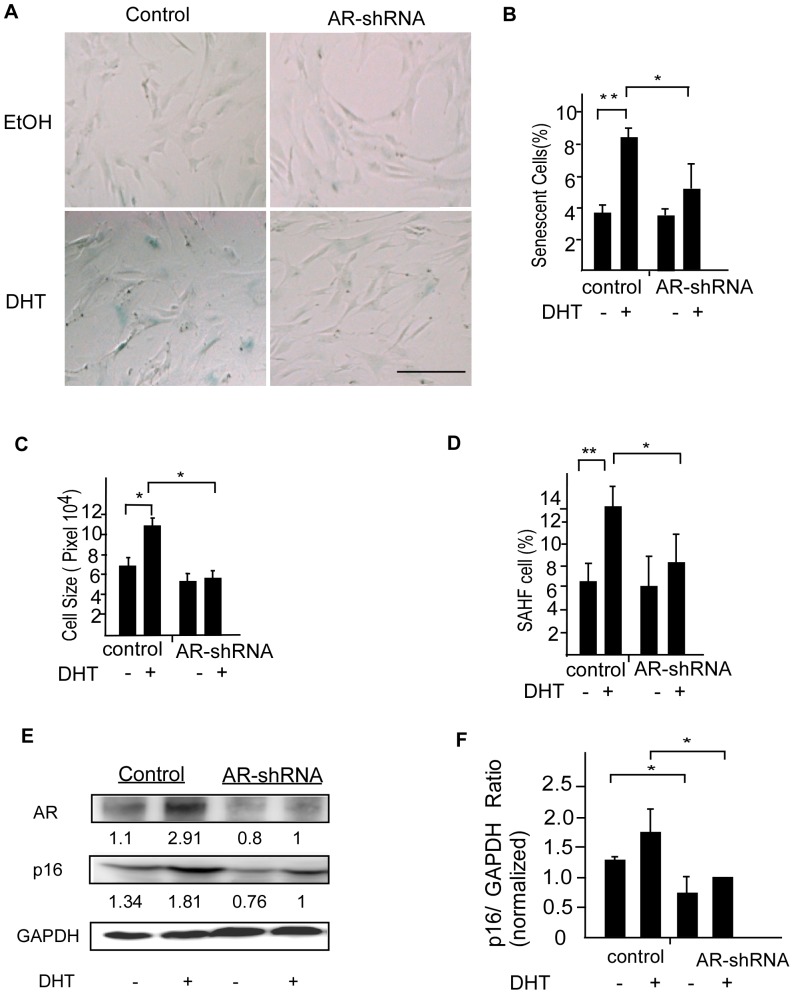
AR knockdown suppresses DHT-mediated senescence in DPCs. (A) DPCs infected with AR-shRNA or scrambled shRNA were cultured in the presence of 0.1 µM of DHT or ethanol for 3 days. Premature senescence of DPCs was evaluated on day 5. The senescence-promoting effect of DHT was inhibited by AR knockdown. Scale bar = 100 µm. (B–D) The percentage of SA-β-Gal activity and Cell size was statistically unchanged in AR-knockdown DPCs after exposure to DHT. DHT induced SAHF formation was suppressed by AR downregulation. Values are means ± SDs from three independent experiments (*P<0.05, **P<0.01). Each data resulted from primary culture cells from 3 individuals and each was measured in triplicates in each cell line. (E) AR knockdown diminished p16*^INK4a^* protein expression. A representative immunoblot was shown. The numbers indicate p16*^INK4a^*/GAPDH and AR/GAPDH ratios. GAPDH was used as an internal standard. (F) Quantitative densitometry of the p16/GAPDH protein expression was shown. Values are means ± SDs from 7 independent experiments. Asterisk indicates *P*<0.05.

To further confirm the role of AR in the induction of premature senescence in DPCs, we knocked down the AR with a lentivirus expressing an AR-specific shRNA and evaluated the senescence-promoting effect of DHT. SA-β-Gal activity assays revealed that knockdown of AR expression led to suppression of DHT-induced premature senescence. AR-knockdown DPCs were more resistant to morphological alteration under androgen-stimulation conditions (Fig. 4A). A quantitative analysis showed that knockdown of AR expression led to suppression of DHT-induced SA-β-Gal activity, premature senescence cell size and the percentage of SAHF-containing cells (Fig. 4B, C and D). Similar results were observed as the expression of p16 protein is significantly decreased in AR-knockdown DPCs, compared with the control cells regardless to DHT treatment (Fig. 4E, F). Taken together, these results show that androgen-induced premature senescence was significantly augmented in DPCs overexpressing AR, and diminished in AR-knockdown cells.

### Androgen/AR Action Leads to DNA Damage in DPCs

**Figure 5:**
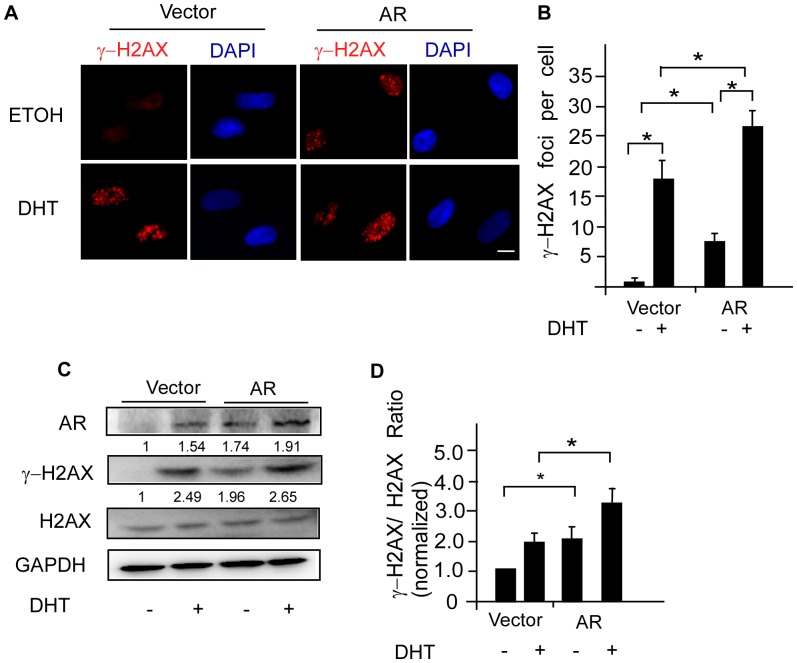
Androgen/AR signaling leads to DNA damage in DPCs. AR was overexpressed in DPCs by transfected with pcDNA3-hAR and cultured in the presence of DHT of 0.1 μM or ethanol for 2 hours. (A) γ-H2AX foci was analyzed by immunofluorescence assays. DHT increased γ-H2AX foci in the nuclei of DPCs; AR overexpression reinforced the effect of DHT. Nuclei were counterstained with DAPI. Scale bar = 10 μm. Foci in cell nuclei were visualized on a microscope using a 40X objective. (B) The mean number of foci per cell was calculated from 40 cells randomly selected for each group. AR overexpression enhanced the statistical significance of DHT effects. Values are means ± SDs from three independent experiments. Asterisk indicates *P*<0.05. (C) A representative immunoblot of cell lysates from DPCs after treatment. The numbers indicate γ-H2AX/H2AX and AR/GAPDH ratios. GAPDH was used as an internal standard. (D) Quantitative densitometry of the γ-H2AX/H2AX protein expression was performed by using ImageJ software. Values are means ± SDs from six independent experiments.

To investigate the DNA damage status after androgen treatment, we transiently expressed the AR in DPCs and employed γ-H2AX as a sensor of double-strained DNA breaks (DSBs). A subsequent immunofluorescence analysis revealed a marked increase in γ-H2AX foci in the nuclei of DPCs after exposure to DHT (Fig. 5A, B). A further increase in γ-H2AX foci (i.e., DSBs) was detected in DPCs stably transfected with the AR upon androgen stimulation (Fig. 5A, B). Moreover, Western blot analyses showed that H2AX had been converted to its phosphorylated form (γ-H2AX) in DPCs in response to DHT, and the γ-H2AX/total H2AX ratio was further increased in cells overexpressing the AR (Fig. 5C,D).

## Discussion

Clinically, patterned hair loss has been reported in non-AGA individuals with iatrogenic androgen stimulation [30], and in patients suffering from diseases characterized by excessive androgen, such as polycystic ovary syndrome and androgen-secreting tumors [31]. It is known that balding DPCs already showed varying degrees of premature senescence in early passages [18]. Moreover, DPCs isolated from non-balding areas of normal individuals have been demonstrated to be androgen-responsive and have been used as a cell model of AGA [32]. AR expression level is a crucial factor in determining DPC sensitivity to androgens in AGA [33]. It is known that AR mRNA [34], [35] and protein [36] are expressed in DPCs. In addition, DPCs isolated from balding area express more AR compared to those in non-balding areas [6], [37]. AR expression is stronger in early-passage DPCs and gradually decreasing during subcultivation [28]. Our results showed that earlier-passage DPCs with higher AR expression were more sensitive to androgen-mediated premature senescence. It is well known that primary DPCs spontaneously undergo replicative senescence during subcultivation. Indeed, we also observed that late-passage DPCs were more senescent (Fig. 2). Loss of responses to androgen-induced senescence in late-passage DPCs could reflect relatively lower AR expression in these cells as well as masking of androgen effects by replicative senescence. In our study, we also demonstrated that androgen-accelerated premature senescence was affected by AR expression level (Fig. 3 and 4). We thus concluded that blocking androgen/AR actions could play an important role in suppressing premature senescence in DPCs.

In prostate cancer cell lines, androgen signaling has been reported to induce recruitment of the AR-topoisomerase II beta (TOP2B) complex, which catalyzes DSBs at regulatory regions of AR target genes [20]. AR also acts in concert with genotoxic stress to induce alterations in local chromatin architecture. These events are permissive for sensitizing these regions to undergo chromosomal translocation through activation of ORF2 endonuclease [38]. In our study, DSBs were also induced in response to androgen/AR signaling, and γ-H2AX foci and expression levels of γ-H2AX proteins were further increased with AR overexpression (Figure 5). These results support previous findings that two important DNA damage sensors involved in the phosphorylation of H2AX–the active form of ATM (ataxia-telangiectasia-mutated kinase) and ATR (ATM and Rad3-related)–were detected only in balding DPCs [18]. Although much of this DNA damage can be repaired and the cell can then re-enter the cell cycle, some of the aberrantly enhanced DSBs might destabilize the genome and potentially trigger premature senescence in DPCs. The roles of TOP2B and ORF2 in DNA damage leading to premature senescence of DPCs need further investigation.

The effects of androgen/AR signaling on senescence in prostate cancer cells remain a matter of controversy [39], [40]. It has been reported that androgen depletion induces senescence in prostate cancer cells via down-regulation of Skp2 [39], [40]. In contrast, androgen/AR signaling has also been reported to drive cellular senescence without the involvement of DNA damage and p16*^INK4a^* upregulation in both prostate cancer and normal immortal prostate epithelial cell lines [39]. In DPCs, we found that p16*^INK4a^* protein levels were upregulated in response to androgen, and AR overexpression may further enhance the expression level of p16*^INK4a^* (Figure 3E). These results suggest that androgen/AR signaling promotes senescence through the p16*^INK4a^* pathway in DPCs and are in agreement with the results of a previous study, which showed increased expression of p16*^INK4a^* in balding DPCs from AGA patients with premature senescence [18]. The discrepant reports of androgen/AR actions in senescence could reflect the diversity of biological responses to androgen/AR signaling in different cell types. While these latter studies utilized the prostate cancer cell lines, PC3 and PC3-AR, and immortalized normal prostate RWPE-1 cells as experimental models, it is well known that PC3 cells are AR-, p16*^INK4a^*- and p53-null [39], and RWPE-1 cells are p53- and Rb-null [41]. Therefore, the senescence response and the pathway that mediates it in these cells might be different from that in primary DPCs. The signaling pathways activated by DNA damage converge on p53/p21 pathway-mediated replicative senescence caused by telomere shortening, and the p16 pathway is thought to mediate premature senescence [19]. Expression of p16*^INK4a^* is induced by numerous stressors, including oxidative stress [42], overexpression of oncogenes [43], [44], and DNA damage [45], [46]. Nuclear expression of oxidative stress and DNA damage markers has been reported in balding DPCs [18]. Increased DSBs and upregulation of p16*^INK4a^* in response to androgen/AR signaling suggest that DNA damage might be important in the androgen-accelerated premature senescence of DPCs, although we cannot exclude the possibility that oxidative stress is also induced by androgen/AR signaling. Androgen inducible molecules, such as Interleukin-6 (IL-6) and transforming growth factor (TGF)-β1 have been shown to inhibit hair growth in paracrine manner [27], [47] and could be contributing factors of cellular senescence. Whether the androgen/AR-accelerated premature senescence of DPCs is also mediated via autocrine manner by androgen inducible IL-6 and TGF-β1 needs more investigation.

The previous studies showed low passage DPCs could sustain epidermal cell proliferation; however, high passage DPCs could not [48]. In addition, DPCs after multiple passaging also reduced hair growth-promoting capabilities *in vivo* [30], [49]. These evidence supports the senescent DPCs may have functional defect on promoting epithelial-mesenchymal interactions. It has been shown that androgen/AR regulates the interaction of DPCs and follicular KCs by androgen-inducible factors secreted from DPCs [27], [28], [47], [50]. Recently, an impairment of hair follicle stem cells to differentiate into progenitor cells was reported to play a key role in the pathogenesis of AGA [51]. Miniaturization of hair follicles, the hallmark of AGA, displays thinner hair fibers and smaller DP size. It is possible that the androgen/AR-induced senescence in DPCs may not only lead to diminished DP size but also deregulate the communication between DPCs and hair follicle stem cells to differentiate to progenitor cells.

Here, we showed a previously unidentified relationship between androgen/AR signaling and induction of premature senescence in association with DNA damage and p16*^INK4a^* upregulation in DPCs. Our study highlights the importance of androgen/AR-accelerated premature senescence in DPCs, a process that is thought to reflect irreversible cell growth arrest in the progression of AGA. The acceleration of premature senescence of DPCs by androgen/AR signaling may explain the miniaturization of hair follicles shown in AGA patients. These results provide novel impacts of androgen/AR signaling in balding DPCs and offer the potential therapeutic targets on combating for AGA.

## Supporting Information

Figure S1 **Estrogen/ERα signaling did not cause premature senescence in DPCs.** (A) Non-balding DPCs of frontal scalp were transfected with pcDNA3-hERα or pcDNA3 empty vector and cultured in the presence of 0.01 µM of 17β-estradiol or ethanol (vehicle control) for 3 days. Premature senescence of DPCs was evaluated on day 5. Scale bar = 100 µm. SA-β-Gal activity (B), cell size (C), and the number of SAHF-containing DPCs (D) were unaltered. Values are means ± SDs from three independent experiments (E) A representative immunoblot of cell lysates of DPCs after treatment with 17β-estradiol or vehicle for 84 hours. The numbers indicate p16*^INK4a^*/GAPDH and ERα/GAPDH ratios. GAPDH (glyceraldehyde 3-phosphate dehydrogenase) was used as an internal standard.
(TIF)
