# Supplementary figures and images for: Androgen Receptor Accelerates Premature Senescence of Human Dermal Papilla Cells in Association with DNA Damage
Source: PLoS One. 2013 Nov 14;8(11):e79434. doi: 10.1371/journal.pone.0079434 (PMC3828374; doi:10.1371/journal.pone.0079434)

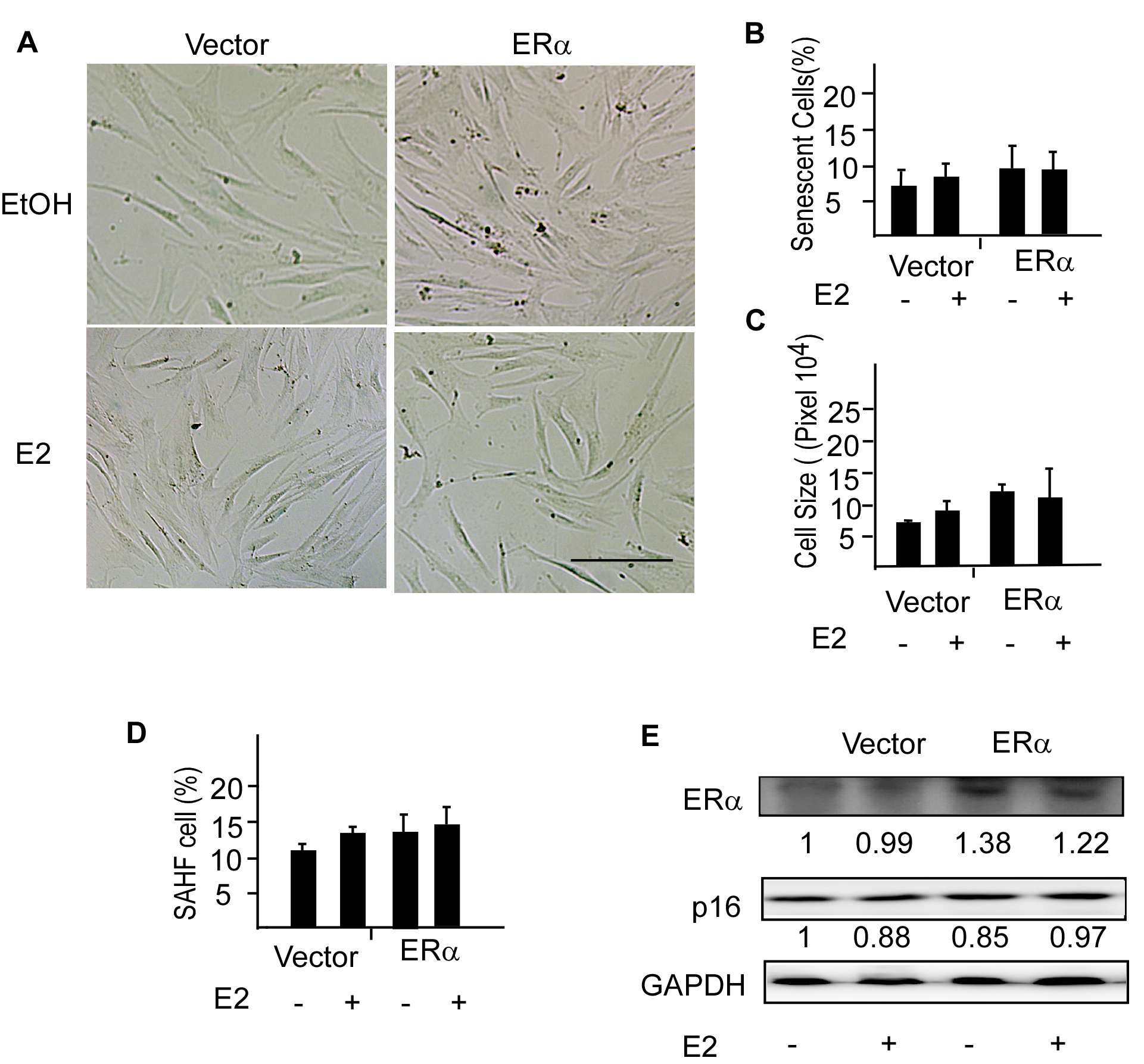

Supplement: Figure S1 — Estrogen/ERα signaling did not cause premature senescence in DPCs. (A) Non-balding DPCs of frontal scalp were transfected with pcDNA3-hERα or pcDNA3 empty vector and cultured in the presence of 0.01 µM of 17β-estradiol or ethanol (vehicle control) for 3 days. Premature senescence of DPCs was evaluated on day 5. Scale bar = 100 µm. SA-β-Gal activity (B), cell size (C), and the number of SAHF-containing DPCs (D) were unaltered. Values are means ± SDs from three independent experiments (E) A representative immunoblot of cell lysates of DPCs after treatment with 17β-estradiol or vehicle for 84 hours. The numbers indicate p16INK4a/GAPDH and ERα/GAPDH ratios. GAPDH (glyceraldehyde 3-phosphate dehydrogenase) was used as an internal standard. (TIF) [file pone.0079434.s001.tif]
